# Future doctors, future scholars: factors influencing China-educated international medical students’ career intentions in primary care and academic medicine

**DOI:** 10.1186/s12960-026-01062-2

**Published:** 2026-03-25

**Authors:** Wen Li, Robyn M. Gillies, Hong Sun, Asaduzzaman Khan

**Affiliations:** 1 School of International Education, Xuzhou Medical University, Xuzhou, 221004 China; 2 School of Education, Faculty of Humanities, Arts and Social Sciences, The University of Queensland, Brisbane, 4072 Australia; 3 School of Basic Medicine, Xuzhou Medical University, Xuzhou, 221004 China; 4 School of Health and Rehabilitation Sciences, Faculty of Health, Medicine and Behavioural Sciences, The University of Queensland, Brisbane, 4072 Australia

## Abstract

**Background:**

Critical workforce shortages in primary care (PC) and academic medicine (AM) persist in many low- and middle-income countries (LMICs). China hosts approximately 68,000 international medical students (IMSs), primarily from LMICs, constituting a potential workforce solution. However, little is known about factors shaping their intentions towards PC and AM. This study investigates these influences using established career-choice frameworks in the Chinese context.

**Methods:**

An exploratory sequential mixed-methods study was conducted following the instrument development model. Qualitative interviews (*n* = 20) identified influencing factors, which informed a quantitative survey distributed to IMSs at 17 Chinese institutions. Responses from LMIC-origin IMSs were analysed. Principal component analysis (PCA) extracted viewpoint components, and hierarchical logistic regression examined the effects of individual, institutional, and viewpoint factors on students’ intentions toward PC and AM.

**Results:**

Qualitative findings revealed IMSs’ career intentions were shaped by multiple interacting factors across home- and host-country contexts. Among the 961 surveyed IMSs, 15.6% (*n* = 150) chose PC specialties, and 36.3% (*n* = 349) preferred AM. PCA identified three components from viewpoint factors: “personal needs to satisfy”, “perceptions of work characteristics”, and “social needs to satisfy” (Kaiser–Meyer–Olkin index 0.946, *p* < 0.001, 60.5% of variance explained). Regression models showed PC preference was positively associated with older age and rural/regional origin, and negatively associated with personal needs to satisfy. AM preference was positively associated with older age, lower study year, originating from the African region compared to other nationalities, and higher-ranked institutions.

**Conclusion:**

IMSs’ intentions towards PC and AM are shaped by student characteristics, personal needs, and institutional environments, offering valuable insights for LMIC workforce planning. Chinese institutions can strengthen this potential by embedding targeted PC and AM modules, expanding research and mentorship opportunities, refining admissions to prioritise likely candidates, and supporting cross-cultural training. These strategies align international medical education with LMIC health priorities and inform career guidance and tailored support for globally trained physicians.

**Supplementary Information:**

The online version contains supplementary material available at 10.1186/s12960-026-01062-2.

## Introduction

In 2006, the World Health Organization (WHO) identified 57 countries—all low- and middle-income countries (LMICs)—with critical health workforce shortages [1]. Nearly two decades later, these deficits persist, compounded by large-scale migration, poor working conditions, alongside new pressures from emerging diseases, climate change, and ageing populations [2]. The shortages are acutely visible in primary care (PC) [3] and academic medicine (AM) [4]. Gaps in these sectors not only weaken frontline service delivery [5], but also erode medical education, research and policy capacity [6], threatening health system resilience, sustainability, and equity [7, 8].

Addressing this crisis requires understanding medical students’ career intentions. However, existing evidence and theoretical models are largely derived from high-income countries (HICs) [9, 10]. Drawing on North American literature, Bland et al. [11] proposed a theoretical model of PC career choice in which specialty selection results from a matching between students’ career needs and their perceptions of specialty characteristics [11]. These needs and perceptions relate to student values and are shaped by individual attributes and institutional features [12]. Querido et al. [12] extended this framework beyond specialty choice to include AM, synthesising evidence from Western European contexts and organising influencing variables into five groups: (1) student attributes (e.g. demographics, background); (2) institutional features (e.g. type, curriculum); (3) needs to satisfy (e.g. altruism, intellectual challenge); (4) perceptions of work characteristics (e.g. status, research opportunities, role models); and (5) student values (e.g. personal priorities). Together, these frameworks align with broader international reviews of PC [9, 13, 14] and AM [15, 16], representing a predominantly Western-derived understanding of medical career choice.

The generalisability of these models to LMIC contexts remains unclear, as pivotal factors may be perceived or weighted differently in under-resourced systems [9, 17]. Existing evidence from LMICs suggests consistently low intentions for PC and AM (e.g. 1.9% chose general practice in Kenya [18], none in Botswana [19]; AM options were least preferred in Bangladesh [20] and India [21]), but these studies remain primarily descriptive and lack conceptual models to explain underlying mechanisms [10].

International medical students (IMSs) represent a promising resource for addressing these workforce shortages. Growing numbers of students from LMICs pursue medical education abroad [22], with China emerging as a major destination, hosting approximately 68,000 IMSs, primarily from LMICs [22]. Most report intentions to return home [23, 24], positioning them as a potentially important contribution to home health systems.

Investigating this cohort is therefore important for both policy and theory. The combination of the Chinese educational environment, students’ status as overseas-trained graduates, and their diverse LMIC origins creates a decision context that Western-derived models may not fully capture. While prior studies have described China-educated IMSs’ career preferences, they did not examine factor-choice associations [25] or apply a theoretical lens [22], limiting mechanistic insight. A theory-informed investigation of factors shaping PC and AM career intentions among LMIC-origin IMSs in China is therefore warranted.

Existing literature and career-choice frameworks indicate that similar individual, institutional, and value-based dimensions shape medical students’ intentions across specialty and academic trajectories [11, 12], providing a unified conceptual basis for examining PC and AM together. Within this framework, PC and AM represent distinct yet complementary career paths [5, 6], which empirically intersect as students navigate trade-offs between PC intentions and academic pathways [26]. Examining PC and AM together therefore enables a nuanced understanding of how students reconcile career values and role expectations.

This study applies Bland [11] and Querido [12] frameworks to examine factors associated with PC and AM intentions among IMSs in China. It contributes to informing policies that better leverage this workforce across their home countries, HICs, and China, where shortages in PC and AM persist, and tests the applicability of Western-derived career-choice theory in a new context, offering insights to support efforts to strengthen PC and AM internationally. This focus aligns with global and national workforce initiatives, including the WHO Primary Health Care Implementation Solutions Initiative [27], PC incentive programmes such as pay-for-performance schemes in LMICs [28], and China’s health system and medical education reforms [29, 30], which prioritise strengthening PC delivery, academic capacity, and workforce leadership. These initiatives underscore the policy relevance of understanding factors influencing PC and AM career intentions.

This study aims to identify key factors shaping professional role and specialty intentions among China-educated IMSs originating from LMICs, and to examine their associations with preferences for PC and AM.

### Research questions

RQ1. What factors are considered by LMIC-origin IMSs educated in China when shaping professional role and specialty intentions?

RQ2. What factors are associated with their career intentions in PC and AM?

## Methods

### Study design

This study employed an exploratory sequential mixed-methods design [31], following the instrument development model described by Schifferdecker and Reed [32]. This approach uses an initial qualitative phase to identify contextually salient constructs and language, which are then translated into survey items for a subsequent quantitative phase [32]. It is particularly well suited to cross-border and under-researched populations, as it enables instruments to be grounded in participants’ lived experiences and sociocultural contexts rather than relying solely on measures developed in high-income or single-country settings. In the present study, the qualitative phase explored factors shaping IMSs’ career intentions (RQ1), and the resulting themes, integrated with relevant literature, informed the development of the survey instrument. The quantitative phase was cross-sectional, using the survey data to examine associations between these factors and PC and AM career preferences (RQ2).

Bland [11] and Querido [12] models guided the entire process. These frameworks were considered suitable because they integrate multi-level variables influencing career choice, encompassing individual, institutional, and value-based influences. This integrative structure offers a more comprehensive approach than single-level or purely motivational models and is well suited to capturing the complexity of career intentions among IMSs in China [22].

### Study context

In China, IMSs typically enrol in 6-year, English-medium medicine programmes [22], comprising 5 years of coursework and a 1-year clinical internship, culminating in a bachelor’s degree in clinical medicine [24]. Curricula are often adapted to health profiles in IMSs’ home countries [33]. IMSs and domestic students attend separate classes but may share hospital rotations [33].

### Ethical considerations

Ethics approval was granted by the Human Research Ethics Committee at The University of Queensland, Australia (2022/HE001071). Permissions to recruit participants were secured from participating universities through designated institutional gatekeepers, who facilitated access to eligible participants in accordance with institutional policies but had no role in consent processes or data collection.

A Participant Information Sheet was provided to all participants. Written informed consent was obtained from interview and focus group participants prior to data collection. For the survey, consent was implied by participants selecting “Yes (to continue on to the survey)” after reading the online information sheet.

Participation was voluntary, and participants were informed of their right to decline participation or withdraw at any time without penalty. Confidentiality was maintained through data deidentification and secure storage on password-protected university servers accessible only to the research team.

### Qualitative phase

#### Participants

Eligible participants were required to be (1) IMSs in their senior years of study (i.e. in their fifth or sixth year), with students in earlier years excluded, as those at this stage typically have greater clinical exposure and more developed career considerations [34], and (2) originating from LMICs, in line with the study aims. To enhance variation in perspective [31, 35], we purposively recruited IMSs from diverse LMIC regions, while also aiming for a balanced gender ratio. Participants were recruited from one medical university hosting IMSs from approximately 40 LMICs, which enabled access to a diverse international student population and facilitated purposeful sampling. Potential participants were approached through WeChat.

#### Data collection

One researcher (WL) conducted online, semi-structured interviews in English with all participants. A self-developed, pilot-tested interview guide, informed by the research questions and relevant literature, was used to explore career plans and influencing factors. Altogether, 20 IMSs were interviewed (age range 21–26, mean 23.75; female/male, *n* = 10/10; 5th-year/6th-year, *n* = 9/11; Asian origin/African origin, *n* = 10/10). We considered the sample adequate, as ongoing analysis indicated that the themes identified provided sufficient depth and variation to address the research questions, consistent with a pragmatic approach to saturation [36].

#### Data analysis

Interviews were audio-recorded, transcribed, anonymised and coded in NVivo (12.6.1). Analysis followed a hybrid thematic analysis as described by Fereday and Muir-Cochrane, combining deductive coding informed by Querido et al.’s conceptual variables with inductive coding to capture context-specific themes beyond existing frameworks [37]. Coding and theme development were conducted iteratively, with reflexivity supported through team discussions and critical examination of assumptions [35, 37]. This process generated a set of empirically derived themes and subthemes supported by illustrative quotations [32]. Subthemes were translated into candidate survey items, drawing on participants’ language where appropriate (Online Appendix 1). These candidate factors were then integrated with broader career choice literature [9, 13–16] to further inform survey item development (Online Appendix 3).

#### Reflectivity and trustworthiness

Interviews were conducted by WL, who has formal training in qualitative methods and research experience in international medical education. Although WL had prior administrative and teaching experience in international medical programmes in China, she had no direct teaching, supervisory, or evaluative relationship with participating IMSs.

The research team comprised scholars with expertise in medical education, health sciences, and international education. Reflexivity was supported through regular team discussions, examining assumptions and exploring alternative interpretations, particularly when findings diverged from expectations. Divergent or negative cases, accounts that differed from majority patterns, were carefully reviewed to refine theme definitions and ensure that the full range of student experiences was represented, consistent with recommended validity practices [38].

Interviews were conducted in English. Potential language or accent-related challenges were mitigated through ongoing comprehension checks, confirmation of interpretations, and the interviewer’s bilingual background, supporting cross-cultural communication. Trustworthiness was further enhanced through the use of a semi-structured interview guide, clarification and paraphrasing during interviews, and iterative team-based discussions during analysis.

### Quantitative phase

#### Sampling and administration

The survey was conducted in 17 institutions across 11 administrative provincial regions: East China (*n* = 8), Southwestern China (*n* = 4), South Central China (*n* = 2), Northeast China (*n* = 2), and Northwestern China (*n* = 1), strategically selected to reflect institutional and regional diversity [22, 33]. Of 30 contacted institutions, 17 approved participation. A non-probability, voluntary response sampling strategy was employed [31], with an open invitation to all students enrolled in international medical programmes at participating institutions. Programme administrators and teaching staff facilitated survey distribution by sharing links in class networking groups and informing students of the study purpose. IMSs across all study years and classes were invited, regardless of country of origin, to ensure clarity of recruitment and maximise reach, although COVID-19-related constraints occasionally limited access. Data were collected anonymously from June to December 2023.

#### Questionnaire design

A questionnaire was developed [32] by directly operationalising: (1) qualitative themes, (2) Bland’s conceptual structure [11], and (3) constructs from Querido et al. [12] and related literature [15–17, 39]. It included three sections: demographics and background; professional and specialty preferences; and factors influencing career intentions (Online Appendix 2).

Aligned with our guiding frameworks [11, 12], we intended to capture key variables across the five groups, adapted to our research context:

##### Student attributes (Group 1 variables)

Collected information included gender, age, year of study, home country, and place of residence. Family background in the medical field was also collected given its relevance to LMIC medical students’ career choices [40].

##### Institution features (Group 2 variables)

Due to limited access to certain model-listed variables (e.g. curriculum specifics), we considered availability for variable selection. Institution type [41], ranking, and local GDP were included for their relevance to PC and AM [42, 43]. These variables reflected model elements, such as admission structure, research emphasis, coursework, and faculty composition [41].

##### Viewpoint variables (Group 3 and 4 variables)

Needs (Group 3) and perceptions (Group 4), regarded as dynamic viewpoint factors [44], were derived from qualitative themes and literature, tailored to IMSs. These were designed as 5-point Likert items from “strongly disagree” to “strongly agree”.

##### Student values (Group 5 variables)

Student values refer to the things they prioritise [11, 12]. As prior research suggests trade-offs between AM and specialty career choices [45, 46], values were operationalised via priority given to an alternate career facet. AM preference served as a control variable when modelling PC preference as the outcome, and vice versa.

##### Career preference measurement

Specialty preference was assessed by asking students to select their single most desired specialty from a list tailored for IMSs in China [22]. Internal medicine and paediatrics were divided into PC and non-PC categories. General practice/family medicine, general internal medicine (PC), and general paediatrics (PC) were classified as PC specialties based on their generalist-oriented roles and alignment with PC in both LMICs and HICs, consistent with the prior study involving LMIC-origin IMSs in China [22]. Professional role preference was measured using a ranking task adapted from a career plan item on the AAMC Medical School Graduation Questionnaire [47]. Students were asked to rank intended professional activities; selecting teaching/research or administration/leadership among the top two options was used to indicate AM preference [6].

#### Questionnaire refinement

The instrument was refined following best practice guidelines [48], incorporating insights from existing literature, expert validation, cognitive interviews, and a pilot study with follow-up focus groups (Online Appendix 3). Three experts in international medical education reviewed the draft, recommending revisions to improve accuracy and parsimony [49]. Cognitive pre-testing with six IMSs ensured response process validity [48]. A pilot at one Chinese university involved 51 IMSs from LMICs. Items with biased distributions or limited response dispersion were identified for further review, as these suggested reduced discrimination among respondents and could benefit from rewording to increase response variability [50]. In addition, written qualitative feedback provided via a comment text box in the pilot survey was also examined. These issues were further explored in focus group discussions with six pilot participants to inform item refinement [50]. This process resulted in the deletion of a single pandemic-related factor, which had also been flagged during cognitive interviews and subsequently supported by the focus group findings (Online Appendix 3). Demographics and career preference items remained unchanged. Curriculum and faculty factors were retained separately to capture institutional characteristics from student perceptions.

#### Statistical analysis

Pilot study participants were instructed not to complete the main survey. Responses from HIC-origin students were excluded. To describe the sample, we compared demographic (individual and institutional) factors for PC and AM preferences using Chi-square tests for categorical independent variables and t-tests for continuous independent variables. Where categorical variables had more than two levels (e.g. student nationality region), overall significant group differences were followed by exploratory post hoc pairwise comparisons to identify which pairs differed in career preferences. Likert-scale viewpoint items were treated as continuous variables, consistent with psychometric research showing that 5-point Likert items can be appropriately analysed using parametric methods such as PCA and t-tests, particularly with moderate to large samples [51]. Independent t-tests compared mean ratings between career choice groups, and principal component analysis (PCA) with Varimax rotation used to identify key components [44]. A total of 17 viewpoint items were entered into the PCA. Prior to PCA, sampling adequacy and factorability were assessed using the Kaiser–Meyer–Olkin (KMO) Measure of Sampling Adequacy and Bartlett’s Test of Sphericity. Components were retained based on eigenvalues (> 1.0), scree plot inspection, and interpretability, with a loading threshold of > 0.40 and cross-loadings lower than the primary loading [44, 52]. Scores of principal components were calculated [53].

Given limited prior knowledge of interrelations between factors [12], we adopted an exploratory approach, using regression models to examine associations and generate preliminary insights [54]. Hierarchical binary logistic regression models examined associations between factors and PC specialty preference: Block 1 (individual attributes), Block 2 (institutional features), and Block 3 (viewpoint components), controlling for AM career preference in each Block. Similar models were built for AM career preference, controlling for PC preference. The hierarchical models were evaluated by examining the incremental contribution of each block using changes in −2 Log Likelihood (−2LL) via likelihood ratio tests, with lower values indicating better model fit. Overall model significance was assessed using the Omnibus Test of Model Coefficients, which tests whether the set of variables improves model fit compared with the intercept-only model; model discrimination was evaluated using the area under the receiver operating characteristic curve (AUC), where 0.5 indicates no discrimination and values closer to 1.00 indicate stronger discriminative performance [55]. Multicollinearity was assessed (VIF < 5.0) [56]. Statistical significance was set at *p* < 0.05. Analyses used IBM SPSS Statistics (Version 29).

## Results

### Qualitative findings

Interviews revealed a broad and interconnected set of factors influencing IMSs’ career intentions, organised into three major themes: personal attributes and motivation, educational influences, and practical considerations and career prospects. Conceptually, these themes mapped onto the core elements of the Bland and Querido frameworks [11, 12], including student attributes, institutional features, career needs, and perceptions of work characteristics. Each theme comprised subthemes and candidate factors (Online Appendix 1). Some factors aligned with established variables in prior models, while others reflected context-specific considerations related to students’ LMIC origins and their experience of studying medicine in China. Factors were often described as interacting.

#### Personal attributes and motivation

Participants frequently described how personal capacities, interests, and values influenced their career intentions, largely aligning with established medical career choice models [11, 12]. *Personal interest* was the most prominently cited influence, with all participants emphasising intrinsic enjoyment when considering future careers. *Altruism* was also common, with nearly half expressing a desire to pursue meaningful socially valuable careers. Perceived *competence* was raised by several participants, who weighed the feasibility of career options against their professional capabilities. *Physical condition* was also mentioned, particularly in relation to the sustainability of physically demanding specialties*.*

Beyond these shared influences, participants highlighted considerations reflecting their LMIC backgrounds. Awareness of *patients’ demand* in home-country health systems shaped career direction with a sense of responsibility. Family and socially related experiences, including *advice from family or friends* and *prior family health problems*, further contextualised career intentions by linking individual preference with broader social obligations.

#### Educational influences

Educational experiences were influential for career thinking. Consistent with prior frameworks, some students highlighted the role of *teachers*, *clinical mentors*, and *role models*, as well as exposure through *clinical rotations* and *school curriculum*, in shaping their understanding of different career paths. Positive interactions with faculty members and inspiring mentors were described as pivotal moments that increased interest in certain specialties or academic pathways.

Meanwhile, several educational influences reflected the distinctive context of IMSs in China. Students noted that specific curricular components, including elective courses and internationalised content, broadened their awareness of career options that had not been previously considered. These experiences were framed as expanding horizons beyond traditional clinical roles available in their home countries, especially for those from resource-constrained health systems.

#### Practical considerations and career prospects

Practical considerations featured prominently in students’ accounts. *Job content* and *work/life balance* were among the most frequently discussed influences, with students evaluating the substance of the work itself and its compatibility with desired lifestyles when considering career options. *Financial reward* was commonly raised, in terms of the sustainability of preferred career paths. Other work-related characteristics, such as *autonomy*, *patient type*, and *work pressure*, were also mentioned, which informed how students assessed the desirability of different career options. Career rewards and future advancement, including *employment opportunities*, *prospects*, and *prestige*, played a role in decision-making as well.

Participants identified contextual factors operating across educational and health systems, including *competition* for positions, access to *further training*, and *gendered* or *media-driven perceptions* of career options. The *COVID-19 pandemic* represented a context-specific influence for IMSs in China, with prolonged interruptions to clinical exposure affecting confidence and shaping career intentions away from clinically intensive pathways.

#### Interplay between factors

Across themes, students often described career decision-making as a process of balancing multiple, interacting influences, reflecting a “matching” between their career needs and work perceptions [11, 12]. For instance, some aligned academic performance with perceived entry requirements: “*You need a certain number of publications to actually rate yourself as an applicant to go into the surgical department*”. Others matched lifestyle needs with workstyle perceptions: “*I want to have that flexibility to be able to go somewhere and still be able to do what I love, which is psychology*”. In other cases, students actively ruled out options where perceived work characteristics mismatched core interests: “*I don’t think I’m interested in the knobs and the connection, so I dropped neurosurgery*”. These accounts illustrate that career intentions were shaped not by single factors in isolation, but through ongoing negotiation among personal attributes and needs, educational experiences, work characteristics, and practical constraints within transnational training pathways.

#### Summary of qualitative phase

Overall, the qualitative phase was exploratory and instrumental. It generated a pool of candidate factors spanning both established variables and context-specific elements, many expressed through interactions. These factors were translated into survey items through an iterative process that mapped qualitative subthemes to constructs specified in the Bland and Querido frameworks. Where themes reflected context-specific experiences of LMIC-origin IMSs, new items were developed to capture these influences. The qualitatively derived items were treated as viewpoint variables and subsequently examined and reorganised empirically via PCA into latent constructs for regression modelling.

### Survey population

Of 981 responses received, 17 from HIC students and three incomplete responses were excluded from the analysis, yielding a final analytic sample of 961 IMSs from LMICs. A response rate could not be calculated due to variability in survey dissemination and uncertainty regarding the total number of students reached during the data collection period. Of the 17 institutions, two contributed fewer than 10 responses, but aggregate reporting and recruitment challenges justified retaining them. Participant characteristics are in Table 1. Gender and nationality distributions matched prior IMS studies in China [22, 24], and university types mirrored the national medical education landscape [22], supporting aggregate-level representativeness.

**Table 1:** Associations of demographic factors with PC specialty and AM career preferences (*n* = 961)

Variables	Subtotal	Specialty preference			Academic medicine preference		
		Choosing a primary care specialty (*n* = 150)	Choosing a non-primary care specialty (*n* = 811)	*p*-value	Ranking teaching /research/leadership as top 2 options (*n* = 349)	Not ranking teaching/research/leadership as top 2 options (*n* = 612)	*p*-value
*Student attribute*							
Gender							
Female	443 (46.1%)	61 (13.8%)	382 (86.2%)	0.146	152 (34.3%)	291 (65.7%)	0.232
Male	518 (53.9%)	89 (17.2%)	429 (82.8%)		197 (38.0%)	321 (62.0%)	
Age (Mean ± SD)	22.96 ± 2.935	23.41 ± 3.370	22.87 ± 2.842	**0.038**	23.64 ± 3.689	22.57 ± 2.318	**< 0.001**
Nationality^a^							
South-East Asia Region	597 (62.1%)	95 (15.9%)	502 (84.1%)	0.759	217 (36.3%)	380 (63.7%)	**0.001**
Eastern Mediterranean Region	245 (25.5%)	35 (14.3%)	210 (85.7%)		72 (29.4%)	173 (70.6%)	
African Region	100 (10.4%)	18 (18.0%)	82 (82.0%)		49 (49.0%)	51 (51.0%)	
Other	19 (2.0%)	2 (10.5%)	17 (89.5%)		11 (57.9%)	8 (42.1%)	
Year of study (mean ± SD)	3.87 ± 1.687	3.87 ± 1.682	3.87 ± 1.689	0.960	3.89 (1.747)	3.86 (1.653)	0.833
Place of origin							
Urban area	482 (50.2%)	59 (12.2%)	423 (87.8%)	**0.004**	187 (38.8%)	295 (61.2%)	0.109
Rural or regional area	479 (49.8%)	91 (19.0%)	388 (81.0%)		162 (33.8%)	317 (66.2%)	
Doctor in family							
Yes	472 (49.1%)	75 (15.9%)	397 (84.1%)	0.814	169 (35.8%)	303 (64.2%)	0.746
No	489 (50.9%)	75 (15.3%)	414 (84.7%)		180 (36.8%)	309 (63.2%)	
*Institution feature*							
Institution type by designated orientation^c^							
Comprehensive university	127 (13.2%)	13 (10.2%)	114 (89.8%)	0.073	49 (38.6%)	78 (61.4%)	0.569
Medicine and Pharmacy university	834 (86.8%)	137 (16.4%)	697 (83.6%)		300 (36.0%)	534 (64.0%)	
Institution ranking^b^		6.83 ± 4.737	6.97 ± 4.915	0.737	5.97 ± 4.836	7.51 ± 4.830	**< 0.001**
Institution location by GDP^d^							
> 1000	481 (50%)	73 (15.2%)	408 (84.8%)	0.712	192 (39.9%)	289 (60.1%)	**0.020**
< 1000	480 (50%)	77 (16.0%)	403 (84.0%)		157 (32.7%)	323 (67.3%)	
Career preference							
Preferring academic medicine	349 (36.3%)	43 (12.3%)	306 (87.7%)	**0.034**	–	–	–
Not preferring academic medicine	612 (63.7%)	107 (17.5%)	505 (82.5%)		–	–	
Choosing a PC specialty	150 (15.6%)	–	–	–	43 (28.7%)	107 (71.3%)	**0.034**
Not choosing a PC specialty	811 (84.4%)	–	–		306 (37.7%)	505 (62.3%)	

^a^Nationality: students’ home countries were grouped according to the World Health Organization country region categorisation via website at https://www.who.int/countries. In our study, South-East Asia Region countries included: Bangladesh, India, Indonesia, Myanma, Nepal, Sri Lanka, Thailand; Eastern Mediterranean Region countries included: Afghanistan, Egypt, Iran, Jordan, Morocco, Pakistan, Syrian Arab Republic, Tunisia, Yemen; African Region countries included: Botswana, Cameroon, Chad, Democratic Republic of the Congo, Ethiopia, Gabon, Gambia, Ghana, Ivory Coast, Kenya, Liberia, Malawi, Mali, Mauritania, Mauritius, Namibia, Nigeria, Republic of the Congo, Sierra Leone, South Africa, Tanzania, Uganda, Zambia, Zimbabwe; Other included European Region (Georgia, Azerbaijan), Region of the Americas (Ecuador, Peru), Western Pacific Region (Cambodia, Fiji, Laos, Malaysia, Papua New Guinea, Philippines, Solomon Islands)

^b^The ranking was arranged within the 17 institutions, according to SHANGHAI RANKING via website at https://www.shanghairanking.cn/rankings/bcur/202410. With the best-rated institution as 1, and last-rated institution as 16 (two institutions tied in ranking places), or in other words, lower values indicated higher ranks

^c^The institution type was grouped according to SHANGHAI RANKING via website at https://www.shanghairanking.cn/rankings/bcur/202410

^d^Billions of GDP in USD in 2023. Reference website https://en.wikipedia.org/wiki/List_of_Chinese_administrative_divisions_by_GDP

^e^Bold values indicate statistically significant associations based on *p*-values (*p* < 0.05)

### PC specialty and AM career preferences and association with student and institution characteristics

Table 1 presents career preferences and associations with student and institution characteristics. Overall, 15.6% (*n* = 150) preferred a PC specialty, more likely among older students (*p* < 0.05) and those from rural/regional backgrounds (*p* < 0.01). By comparison, 36.3% (*n* = 349) preferred an AM career, more likely to be older (*p* < 0.001), from higher-ranked institutions (*p* < 0.001) and in higher GDP places (*p* < 0.05). Exploratory post hoc tests indicated students from the African Region were more likely to prefer AM than those from the Eastern Mediterranean Region (*p* < 0.05). Students preferring AM were more likely to choose non-PC specialties (*p* < 0.05), and those selecting PC were less likely to indicate AM preference (*p* < 0.05).

### Viewpoint factors comparison and PCA results

Online Appendix 4 shows comparisons of viewpoint factors. PC-preferring students rated personal interest, study content and environment, and salary/financial reward significantly lower than non-PC peers. AM-preferring students rated personal interest significantly higher.

PCA identified three components (60.5% of variance explained) (Table 2). The KMO Measure was 0.946, and Bartlett’s Test of Sphericity was significant (*p* < 0.001), confirming data suitability [44]. Components were labelled based on content and models [11, 12]: (1) “personal needs to satisfy”; (2) “perceptions of work characteristics”; (3) “social needs to satisfy”.

**Table 2:** Items of students’ viewpoint factors using principal component analysis for extraction and a Varimax rotation

Items	Component		
	1	2	3
Personal interest	**.835**	.217	.068
Competence	**.777**	.211	.215
Altruism	**.774**	.195	.114
Physical condition	**.670**	.205	.327
Studying content and environment	**.563**	.364	.421
Work content and environment	**.496**	.438	.430
Employment opportunities	.335	**.701**	.082
Salary/financial reward	.374	**.694**	.160
Competition	.259	**.675**	.234
Prestige	.217	**.669**	.237
Gender representation gap	− .061	**.660**	.257
Career progression outlook	.569	**.626**	.105
Work/Life balance	.434	**.520**	.245
Role model	.315	**.433**	.411
Previous or existing health problems in the family	.070	.124	**.749**
Advice from family, friends or peers	.160	.240	**.681**
Teachers or mentors at school or hospital	.410	.272	**.620**

	C1	C2	C3
	*% Variance explained*		
	24.065	22.376	14.075
	*Component label*		
	Personal needs to satisfy	Perceptions of work characteristics	Social needs to satisfy

The most salient loadings are shown in bold

### Hierarchical regression analysis

**Fig. 1:**
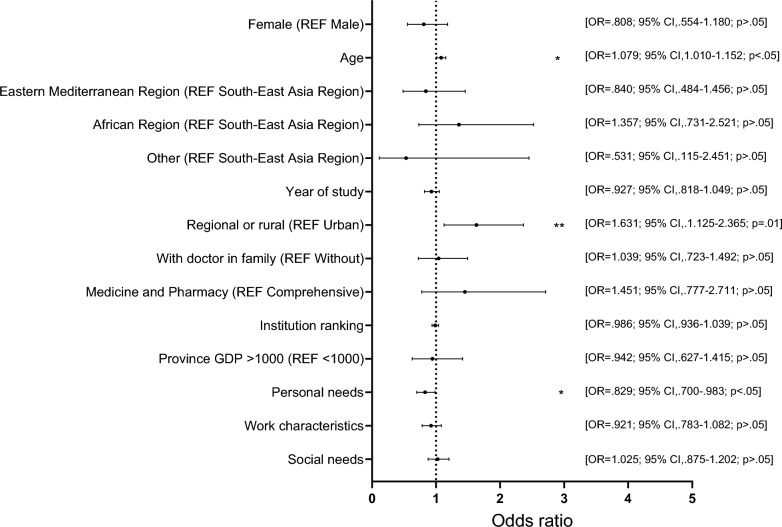
Factors associated with primary care specialty preference among international medical students in China, showing ORs with 95% CIs from the final hierarchical logistic regression model (Model 3; data from 17 Chinese institutions, June–December 2023). The model included student attributes (gender, age, nationality, year of study, place of origin, doctor in family), institution features (institution type by designated orientation, institution ranking, and province GDP), and viewpoint factors (personal needs to satisfy, perceptions of work characteristics, and social needs to satisfy), while controlling for AM career preference. Statistical significance was set at *p* < 0.05. Full regression outputs are in Online Appendix 5. Abbreviations: *CI* confidence interval; *IMS* international medical student; *OR* odds ratio

**Fig. 2:**
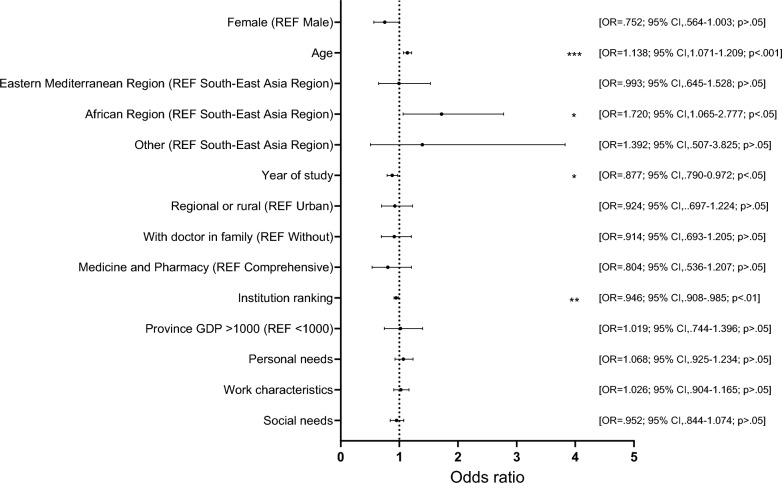
Factors associated with academic medicine career preference among international medical students in China, showing ORs with 95% CIs from the final hierarchical logistic regression model (Model 3; data from 17 Chinese institutions, June–December 2023). The model included student attributes (gender, age, nationality, year of study, place of origin, doctor in family), institution features (institution type by designated orientation, institution ranking, and province GDP), and viewpoint factors (personal needs to satisfy, perceptions of work characteristics, and social needs to satisfy), while controlling for PC specialty preference. Statistical significance was set at *p* < 0.05. Full regression outputs are in Online Appendix 5. Abbreviations: *CI*, confidence interval; *IMS*, international medical student; *OR*, odds ratio

Table 3 displays results from three hierarchical logistic regression models for PC and AM preferences: Model 1 only included students’ attributes, Model 2 added institution features, and Model 3 added to model 2 viewpoints. For both outcomes, −2LL values decreased across successive blocks, indicating incremental improvements in model fit. Specifically, for PC, −2LL values decreased from 808.465 (Model 1) to 806.699 (Model 2), and further to 801.166 (Model 3). For AM, −2LL values decreased from 1205.267 (Model 1) to 1194.274 (Model 2), and to 1192.705 (Model 3). Figures 1 and 2 show the odds ratios (ORs) of each variable with respective 95% confidence intervals (CIs) in the final Model (Model 3). Online Appendix 5 provides full details.

**Table 3:** Hierarchical regression models of PC specialty and AM career preferences

	PC specialty preference			AM career preference		
	Model 1	Model 2	Model 3	Model 1	Model 2	Model 3
	B (S. E.)	B (S. E.)	B (S.E.)	B (S. E.)	B (S. E.)	B (S. E.)
*Student attributes*						
Gender						
Female (REF Male)	− 0.212 (0.191)	− 0.211 (0.192)	− 0.213 (0.193)	− 0.237 (0.145)	− 0.288 (0.146)*	− 0.285 (0.147)
Age	0.085 (0.032)**	0.077 (0.033)*	0.076 (0.033)*	0.143 (0.029)***	0.129 (0.031)***	0.129 (0.031)***
Nationality						
Eastern Mediterranean Region (REF South-East Asia Region)	− 0.250 (0.238)	− 0.179 (0.279)	− 0.174 (0.281)	− 0.334 (0.184)	0.004 (0.219)	− 0.007 (0.220)
African Region (REF South-East Asia Region)	0.234 (0.303)	− 0.249 (0.311)	− 0.306 (0.316)	0.425 (0.233)	0.562 (0.242)*	0.542 (0.244)*
Other (REF South-East Asia Region)	− 0.595 (0.777)	− 0.623 (0.780)	− 0.632 (0.780)	0.515 (0.509)	0.327 (0.511)	0.331 (0.516)
Year of study	− 0.086 (0.062)	− 0.070 (0.063)	− 0.076 (0.064)	− 0.112 (0.050)*	− 0.131 (0.053)*	− 0.132 (0.053)*
Place of origin						
Regional or rural (REF Urban)	0.528 (0.187)**	0.513 (0.189)**	0.489 (0.190)**	− 0.141 (0.141)	− 0.085 (0.143)	− 0.079 (0.144)
Doctor in family						
Yes (REF No)	0.006 (0.183)	− 0.012 (0.184)	0.038 (0.185)	− 0.089 (0.140)	− 0.082 (0.141)	− 0.090 (0.141)
*Institution features*						
Institution type by designated orientation						
Medicine and Pharmacy (REF Comprehensive)		0.386 (0.317)	0.372 (0.319)	–	− 0.224 (0.207)	− 0.218 (0.207)
Institution ranking	–	− 0.012 (0.026)	− 0.014 (0.027)	–	− 0.056 (0.021)**	− 0.056 (0.021)**
Province GDP						
> 1000 (REF < 1000)	–	− 0.069 (0.206)	− 0.059 (0.208)	–	0.031 (0.160)	0.019 (0.161)
Needs and perceptions						
Component 1 (Personal needs to satisfy)	–	–	− 0.187 (0.087)*	–	–	0.066 (0.073)
Component 2 (Perceptions of work characteristics)	–	–	− 0.083 (0.082)	–	–	0.026 (0.065)
Component 3 (Social needs to satisfy)	–	–	0.025 (0.081)	–	–	− 0.049 (0.061)
Control						
Choosing a primary care specialty	–	–	–	− 0.526 (0.204)**	− 0.519 (0.205)*	− 0.500 (0.206)*
Preferring academic medicine	− 0.539 (0.206)**	− 0.537 (0.207)**	− 0.513 (0.207)*	–	–	–
Likelihood ratio test						
− 2 Log Likelihood (−2LL)	808.465	806.699	801.166	1205.267	1194.274	1192.705

*P < 0.05; **P ≤ 0.01; ***P ≤ 0.001

The final PC and AM Models were statistically significant, as indicated by the Omnibus Test of Model Coefficients (PC: *χ*^2^ = 25.767, *p* = 0.012; AM: *χ*^2^ = 65.053, *p* < 0.001), and showed fair discrimination with AUC values of 0.632 for PC and 0.641 for AM.

#### PC specialty preference

In Model 1, age and place of origin were significantly associated with PC specialty preference. Controlling for Model 1, no institution features were significant in Model 2. In Model 3 (Fig. 1), adding viewpoint factors revealed that age (OR 1.08, 95% CI 1.01–1.15) increased odds of PC preference, while “personal needs to satisfy” (OR 0.83, CI 0.70–0.98) decreased odds. Place of origin demonstrated the strongest association, with regional or rural origin raising odds of PC preference (OR 1.63, CI 1.13–2.37).

#### AM career preference

In Model 1, age and year of study were significantly related to AM preference. Controlling for student attributes, institution ranking was significantly associated with AM preference; meanwhile, with institution factors added, gender and nationality became significant. In Model 3 (Fig. 2), age (OR 1.14, 95% CI 1.07–1.21) increased the odds of AM preference, while year of study (OR 0.88, CI 0.79–0.97) and institution ranking (lower values indicating higher ranks) (OR 0.95, CI 0.91–0.99) decreased the odds. Nationality demonstrated the strongest correlation, with African origin increasing the odds of AM preference (OR 1.72, CI 1.07–2.78). Moreover, with viewpoint factors added in Model 3, gender was no longer significant.

## Discussion

This mixed-methods study, following an instrument development model, provides a theory-informed account of career intentions among China-educated IMSs from LMICs. By testing universal and context-specific factors derived from interviews, our findings support the relevance and core architecture of Bland and Querido frameworks [11, 12], demonstrating their applicability in a novel population. The three PCA-identified components (personal needs, social needs, and work perceptions) map closely onto the frameworks’ central constructs of career needs and career perceptions. Crucially, our findings clarify how career needs may be differentiated into personal and social dimensions in transnational training contexts, and illustrate the matching premise [11, 12] between needs and perceptions through convergent qualitative and quantitative evidence (changes in variable significance across regression models).

The regression models demonstrated fair discriminative ability (AUC > 0.60 for both PC and AM), indicating that framework-derived factors are meaningfully associated with career intentions. This level of discrimination aligns with cross-sectional studies of complex behavioural intentions [57]. However, the fair but limited discriminative accuracy also clarifies the frameworks’ explanatory boundaries for this population. The decreased −2LL values across hierarchical models reflected modest but incremental improvements in fit, in line with the theory-driven structure examining the sequential contributions of conceptually distinct variable sets. While findings support the exploratory aim of testing established theoretical variables in a new context, they also point to the need for incorporating more granular, context-specific factors in future model refinement. Overall, the frameworks provide a useful starting point for understanding career intentions among LMIC-origin IMSs, while underscoring the importance of context-sensitive interpretation when applying these models across global training environments.

Our findings show distinct patterns when compared internationally. The PC preference rate (15.6%) was similar to a prior China-based study [22], higher than rates reported in Nepal (1.2%) [40] and Angola (3.8%) [58], but lower than in South Africa (34%) [59]. Qualitative findings contextualise this intermediate position. In interviews, participants described strategically orienting towards less competitive specialties, including family medicine, in response to perceived disadvantages faced by overseas-trained graduates in accessing highly competitive residency pathways. This strategic navigation helps explain why PC may attract interest among IMSs in China despite the country’s early-stage referral system and limited structured PC training [22]. Similar patterns have been observed among abroad-educated Canadian physicians, who also report selecting less competitive specialties to facilitate workforce entry [60].

In contrast, the AM preference rate (36.3%) was substantially higher than in other LMIC contexts, such as Malawi (nil immediate, 12% future) [61] or Gambia (nil basic science preference) [62]. A likely explanatory factor is China’s institutional environment, particularly the “New Medical Education” reform initiative fostering research, innovation, and leadership [30]. Qualitative accounts support this interpretation, with students valuing research engagement, scholarly rigour, and learning beyond examination-oriented training. This supportive academic climate appears to elevate AM aspirations among IMSs, highlighting their potential to strengthen home-country academic capacity [2, 63].

Students’ characteristics were influential. Older age and regional/rural origin were linked to PC preference, consistent with global literature [64–66]. AM preference correlated positively with age but negatively with study year, possibly reflecting flexible age criteria for IMS admissions and the burden of clinical training on research time for clinical-year students [15], a tension also reflected in interview accounts where students contrasted exam- and ward-focused training with aspirations for research-oriented learning. These insights inform admission and curriculum strategies.

The negative association between personal needs and PC preference warrants careful interpretation. In this study, the personal needs component comprised personal interest, perceived competence, altruism, physical condition, and perceptions of studying and work environments. While PC is often framed as aligning with altruistic motivation, our findings suggest that in contexts with limited PC training and weak institutional support [33], PC may be perceived as less able to satisfy multiple personal needs simultaneously. Prior studies similarly report that students may view PC as offering fewer opportunities for intellectual engagement, skill development, and supportive learning environments [67, 68]. Qualitative interviews provided indirect support for this interpretation; participants frequently described choosing other specialties because they are “deeper”, “challenging”, “exciting” or full of “adrenaline”. Their emphases on depth, challenge, and intensity help contextualise why PC may be perceived as less aligned with these personal needs within the current training environment.

Curricular and experiential factors therefore played an important role in shaping PC intentions. As shown in Online Appendix 4, ratings of “Studying content and environment” differed significantly between PC- and non-PC-preferring students, highlighting how pedagogy and educational experiences can influence career paths. To promote PC preferences, institutions could enhance PC-relevant curricula through dedicated modules, clinical rotations in underserved settings, and LMIC-enhanced electives addressing priorities such as infectious disease control and chronic care. In contrast, supporting AM pathways may require earlier research exposure, structured mentorship, and informal teaching opportunities [69]. This aligns with students’ expressed desire for institutional support mechanisms that facilitate research engagement beyond licensing preparation during interviews. Cross-cultural educational dialogues co-designed with IMSs may further align training experiences with home-country health system needs.

Notably, IMSs from African regions exhibited stronger AM intentions, mirroring trends among Black/African American medical students [70]. China’s emphasis on research and advanced technologies equips IMSs with skills often lacking in African institutions due to educational and infrastructure gaps [63], enabling their meaningful contributions upon return, particularly through research, teaching, and system-level leadership. Strengthening China–Africa institutional partnerships through placements and collaborative research could further leverage this potential.

Post-pandemic transformations in medical education emphasise the need for physicians skilled in PC and innovation [71]. China’s enhanced training in scientific inquiry, digital health, and public health emergency preparedness [30] may help meet this demand, bridging gaps in routine care and crisis response in LMICs.

### Limitations

The study has limitations. First, the qualitative sample was drawn from a single institution, which may limit institutional diversity and the transferability of findings. Inclusion of multiple institutions in future studies could provide additional qualitative perspectives. Second, its cross-sectional design limited causal inference, and the observed associations should not be interpreted as predictive of future career behaviours; longitudinal studies are recommended to examine temporal and causal relationships [11, 12]. Third, career intentions were self-reported and may not necessarily translate into actual career outcomes. Fourth, respondents selected one most desirable specialty, though real-world choices may be multiple. Fifth, survey-period constraints, including COVID-19-related disruptions and voluntary participation, prevented calculation of a precise response rate. This may introduce selection bias and limit generalisability; future studies conducted under normal conditions, with calculable response rates, could validate and extend these findings.

While the Bland and Querido frameworks guided this study, these Western-derived models may not fully capture contextual and cultural nuances relevant to a cross-border LMIC-origin population, potentially affecting their applicability. Moreover, although pilot study participants were instructed not to complete the main survey to minimise potential priming effects, compliance could not be independently verified due to the anonymous survey design. Institutional variables were constrained by accessibility of data; while proxies (e.g. ranking, GDP) and student-reported perceptions were used, relevant features may have been omitted; direct institutional data could be incorporated to strengthen future work. In addition, given that participants were nested within 17 institutions, potential institution-level clustering effects may have contributed to the observed associations between variables. Finally, as the interviewer and participants are from different cultural backgrounds and may not be native English speakers, language-related limitations could have affected the depth of insights obtained from the qualitative interviews.

## Conclusions

This study examined factors associated with professional role and specialty intentions among China-educated IMSs from LMICs, focusing on PC and AM preferences. It extends Bland and Querido’s career-choice frameworks by differentiating personal and social career needs and illustrating their applicability in a transnational LMIC context, while highlighting how needs and perceptions converge to influence career intentions. Student characteristics, personal needs, and institutional environments meaningfully influenced PC and AM intentions, offering insights for admissions and curriculum design. Targeted PC and AM modules, clinical rotations, research opportunities, and cross-cultural dialogues can better align training with LMIC health priorities.

Building on this study’s exploratory findings, future research could use longitudinal designs and structural equation modelling (SEM) to examine causal pathways linking different dimensions to career intentions, tracking how PC or AM preferences evolve into actual careers and identifying key facilitators and barriers. Extending this work to LMIC-origin students in other countries or international trainees from diverse backgrounds would allow testing and refinement of the Bland and Querido frameworks. Further studies could guide the development of targeted career guidance, mentoring, and tailored support, incorporating context-specific factors such as institutional policies, diaspora networks, and licensing realities, generating evidence to support globally trained physicians in pursuing health system-aligned careers.

## Supplementary Information

Additional file1 (DOCX 31 KB)

Additional file2 (DOCX 28 KB)

Additional file3 (DOCX 21 KB)

Additional file4 (DOCX 25 KB)

Additional file5 (DOCX 26 KB)

## Data Availability

The data that support the findings of this study are available on request from the corresponding author. The data are not publicly available due to privacy or ethical restrictions.

## Abbreviations

AM: Academic medicine
HIC: High-income countries
LMIC: Low- and middle-income countries
IMS: International medical students
PC: Primary care
PCA: Principal component analysis
