# Supplementary material for: Future doctors, future scholars: factors influencing China-educated international medical students’ career intentions in primary care and academic medicine
Source: Hum Resour Health. 2026 Mar 25;24:20. doi: 10.1186/s12960-026-01062-2 (PMC13137620; doi:10.1186/s12960-026-01062-2)
Supplement: Supplementary file 1 — Additional file1 (DOCX 31 KB) [file 12960_2026_1062_MOESM1_ESM.docx]

**Appendix 1** Qualitative themes, subthemes, and illustrative quotations supporting candidate factors influencing career intentions among IMSs

| **Theme** | **Subtheme** | **Candidate factor** | **Illustrative quotations from qualitative interviews** |
| --- | --- | --- | --- |
| Personal attributes and motivation | Health and professional capacity | Physical condition | - So she was like a woman goes through periods, pregnancy, and stuff, too much become like sensitive to blood so that’s not like the best option for a woman. |
|  |  | Competence | - Because to get internal medicine in my home country, you need to have very high score in your entrance examination. - Here in my country, it’s a huge plus, if you’re able to do both the physician’s job as a doctor, and if you have some ability to do some administrative work. |
|  | Personal interest | Personal interest | - And obviously, for the paediatrics, it’s a child specialist, I love for children. So that being my personal interests, so I chose this also. - So, I’m very interested in health care policy and influencing health care policy and, basically that side medical practice really intrigues me. - My plan is like a really big plan. Not only in my home country, I want to open hospitals in all parts of the world. I think it’s a bit funny, but it’s my dream. |
|  | Personal values | Altruism | - There’s no paediatrician in my area, so I want to do my job there to help children there - Yes, they improve policy making, basically, so it tries to influence people who are in government positions to make laws and policies that are good for healthcare. That will benefit people. - No, I’m not telling we have only in our country, there are a lot of poor people all over the world. So that’s why once my hospital grows bigger and bigger, I want to open my hospital branches all over the world. |
|  |  | Patients’ demand | - That's why I think my country needs more paediatrics. So that it will help the healthcare - Because there’re too many heart patients in my home country. - In my country, at least, we are not very forward in terms of sexual education, or sex adults, things like this, and I feel a lot of youngsters, adults, speak of judgements when they go to a gynaecologist, and that is something I can do, that I can be better, I’m not saying in terms of practice and they are very good amazing doctors, but I feel I’ve heard from a lot of my friends, that we’ve gone there for the first time they feel a little bit challenged, in terms of sex or things like that - So, the other NGO, there’s, I mean, an NGO quarter in my country. They specifically deal with HIV and AIDS, trying to create awareness and sensitize people, to reduce the rates of increasing AIDS infections in my country, and help even the people who already have AIDS get drugs and get tested, so I feel like they also would be willing to take on a fresh graduate as a volunteer for such. |
|  | Family and social influences | Advice from family | - My family support me to be a gynaecologist. |
|  |  | Advice from friends | - It can be a friend here in my class, it can be a senior in school, who gives me advice - I have my friends also, who will support me. |
|  |  | Previous or existing health problems in the family | - My mom used to have a lot of complications with her uterus, and you know, even when I was born she told me, how she had a lot of miscarriages and whatever, and one of the gynaecologists that she used to see helped her to, then you know, have a successful pregnancy, up until she carried me to term, so yeah that made me fall in love with gynaecology and obstetrics. - Actually, this is related to my family members. My grandfather, he died because he was having a kidney disease, he got kidney failure. Yeah, so little bit, I have some emotionally attached to it. |
| Educational influences | Mentorship and role models | Teachers and faculties | - I think a teacher, she taught us cardiology. Yeah and I just really liked the way she taught us, and she made me fall in love with it as well, I think she really taught it so well and explained concepts really well, so I really fell in love with it at that point, and I said, oh, I never thought I would fall in love doing it, because, when I was in second year doing cardiovascular physiology, I just thought like cardiology was too long, but yeah and now it’s something that I definitely look forward to doing. |
|  |  | Clinical mentors | - It can be a doctor in hospital, as I’m doing an internship right now, so they really do have an effect - And yeah, other medical professionals have also had some advice |
|  |  | Role model | - Okay, I would say, I have met good cardiologists, of course. At the moment, I’m in the paediatric ward, but I’ve actually met a very good paediatric cardiac surgeon, and I learned a lot from him, so I admire him, and I think it does make me want to be more like him, so, yeah, I guess, I would say I made my choice on the cardiologists I have met. - He’s from a foreign neighbouring country, and that teacher he’s a motivational speaker, and he motivated me a lot. Like he told me like, don’t just work only for you, like you work, you get money, your family. Work for the whole community. So, he gave me this idea, and he motivated me. Work not only for yourself. If you are a doctor, you can save a lot of patients. And don’t keep the knowledge only with you, spread your knowledge. So that’s why I got this idea from him that I want to work and treat the patients and on, I also want to teach the students also, because I want my knowledge to go on and on and on and on, not just stop from me. |
|  | Curriculum and Training | School curriculum | - When we were learning, I took an elective in philosophy, and then we also had the psychology course, that was part of the MBBS program, and the psychiatry course, and I found that those courses, they impacted me differently, gave me a different perspective on life. And so, I’d like to develop that aspect of my life as well. - When I was in China, last year actually when I was in fourth year, I did an elective, Global Health, and that really opened my mind to global health as a career opportunity for the not-so-distant future. |
|  |  | Clinical rotations | - I’m mostly focusing on experience and what it is, I go through every experience, so I’m going to experience every department and it’ll depend on where I have the most fun and I feel like I’m enjoying work the most. So far, it has been surgery. So that’s my choice. - All healthcare sectors need to improve, but I personally think that paediatrics is in a very bad position. |
| Practical considerations and career prospects | Job content and work environment | Job content | - Trauma surgery is like general surgery, and you get to work on different body parts. You do the surgeries, you save people’s lives in acute situations. - Other specialties like surgery, I think it’s deeper… - I still like the whole adrenaline and saving lives thing… - I like to challenge myself continuously… - And the primary priority why I choose the neurology is neural system always excited me… - So, from what I learned about global health, while I was in China, the main work that people who practice what we’ll help do is influence healthcare policy, change in the making of health care policies or policies that can influence the improvement or betterment of health care in different countries and different communities, and they do that without trying to impose. - I want to teach the students, and then they will teach the students, they will teach. I want the knowledge to be spreading. |
|  |  | Work pressure | - If anything happens then it’s all responsible for the surgeon. So, I don’t want to take a lot of stress, and a lot of pressure. |
|  |  | Patient type | - My first choice is internal medicine, because when I do my internal medicine, they will teach both. Like I will know about the adults and also, I can know about the kids also. But if I do paediatrics, I have to be concerned only about the kids. That's the reason which I took my first preference as internal medicine. |
|  |  | Autonomy at work | - One led me to choosing psychology as well, was the fact that, you know, you can do therapy online as well. You don’t necessarily have to be in person to do it. - And I don’t like working under somebody, I don’t want to work under someone, I want to have my own business, and I want to work as myself. Also, that’s the reason I choose to open my own hospital and be the boss, not the worker. - Global health presents me with a nice opportunity to feed the interest I have in helping good laws be enacted, and policies that would help impact people’s lives positively, and the best field you can do that is health care, because if you help your population get healthier, you help your people get healthier, you are going to help the economy, you influence everything through health. |
|  |  | Work/Life balance | - I can’t take a job that’s going to take almost 24 hours a day. |
|  | Career rewards and prospects | Prestige | - I would say, towards cardiology because firstly it’s a very respected profession. - I would like to go outside medical practice, that would feed that zeal that I have to influence policy and help lawmakers make good laws. - And then how marketable they are, like I said, some jobs are not really looked at as highly as others here |
|  |  | Employment opportunities | - There’re a lot of job opportunities for orthopaedic surgeon. - There’s a lot of non-government organizations around that I’m sure would accept me to volunteer with them, and then, hopefully, if I do get employed, let’s say, a year after next year, a year after I write my license exams... |
|  |  | Career prospects | - Main reason was psychology isn’t really a career back home. - I would like to, with global health and how they focus on health care policy making, I feel like, if I do, in the future, want to invest more in my interest in politics, global health, plus my background in medicine combined, can help me advance in a political career, that’s specifically for medicine. So, influencing health care policy in my country. |
|  |  | Salary/Financial reward | - Orthopaedics, because of the salary thing. I would go with the salary thing. They give handsome salary package to orthopaedics and paediatrics as well. - What you use your money for is up to you, but I’m planning to, non to stay forever, just like in the hospital, I would like to set up a few businesses, medical oriented like the hospital. |
|  | Contextual factors | Media | - But I do watch a lot of medical series, medical shows, everything I can find on TV, such as Grey’s Anatomy, Chicago MED. So, when I do that, I obviously get exposed to characters who are involved in different fields, and the characters that are most drawn to other ones, who are participating are orthopaedic surgery. |
|  |  | Further training | - Then the time taken to finish up something. If I need like 16 more years to reach somewhere, I think it will be a bit different from something that requires 6 more years from now, because I’ve already spent almost 1/3 of my life in studying and I’m ready to spend another 1/3 just, you know, to finish up things. |
|  |  | Gender imbalance | - Like obstetrics and gynaecology has more women and neurology has more men. |
|  |  | Competition | - If I don’t get internal medicine, maybe I will choose paediatrics, about children. You will get the seat very easily. - So, looking for a residency programme in those kinds of countries is very competitive, and those attractive positions like orthopaedics, the attracting medical positions will be filled by mostly the doctors that are trained in their countries, people that went to school in their countries. Their residents, their citizens. So, to join in the workforce in their medical programmes, I would have to go for a less competitive specialisation while also doing what I love. If I just want like a less competitive position, maybe I’ll decide on going into family medicine, or pathology, or something like that. |
|  |  | Pandemic | - And the speciality, I was always interested in neurology, but after the COVID-19 pandemic, because of the COVID-19 pandemic, I was back to my country. I didn’t go to the practical classes, clinical process as I needed. So, I’m afraid to take mainstream clinical subject. - So, the sides for pandemics, how to deal with them, how to manage disasters like the COVID pandemic itself, and other disasters that can affect people’s health. I think that's they really actually contributed to me deciding to go into global health. There’s a lot of disaster management, I think, in the profession, and disaster preparedness. |
