# Supplementary material for: Future doctors, future scholars: factors influencing China-educated international medical students’ career intentions in primary care and academic medicine
Source: Hum Resour Health. 2026 Mar 25;24:20. doi: 10.1186/s12960-026-01062-2 (PMC13137620; doi:10.1186/s12960-026-01062-2)
Supplement: Supplementary file 2 — Additional file2 (DOCX 28 KB) [file 12960_2026_1062_MOESM2_ESM.docx]

**Appendix 2** Questionnaire

This questionnaire seeks information from international medical students studying in China on their opinions about career decision making, so as to help students make more informed career decisions. The information will also be used for research on how to develop and improve career guidance service for international medical students in China. Participation in this survey is voluntary. The answers you give will be kept private. Thank you for your participation.

1. Please read the attached Participant Information Sheet (Survey), and indicate your answer in the following statement:

I have read and understood Participant Information Sheet (Survey) and agree to participate. By continuing with this survey, I grant permission to share my responses in the confidential manner described in the Participant Information Sheet (Survey).

| ○Yes (to continue on to the survey) |
| --- |
| ○No |

1. You are a student from _______.

| ○University A |
| --- |
| ○University B |
| ○…… |

1. Your gender _______.

| ○Male |
| --- |
| ○Female |
| ○Other (Please specify) _________________ |

1. Your age _______.
2. Your year of study _______.

| ○Year 1 |
| --- |
| ○Year 2 |
| ○Year 3 |
| ○Year 4 |
| ○Year 5 |
| ○Year 6/Internship year |

1. Your home country ________
2. Your place of residence in home country _________

| ○Rural area |
| --- |
| ○Semi-urban (regional) area |
| ○Urban area |

1. Do you have any family member who works in medical field as a doctor?

| ○Yes |
| --- |
| ○No |

1. Profession and specialty intention

(1) In which of the following activities do you plan to participate during your career? Please rank the top 3 activities according to your preference.

| [ ] Patient care |
| --- |
| [ ] Public health |
| [ ] Teaching/Research |
| [ ] Administration/Leadership |
| [ ] Other (please specify) |

(2) When thinking about your medical career, what is your most desired specialty?

| ○General practice/Family medicine |
| --- |
| ○Medicine specialties (e.g. internal medicine, neurology, etc) (Please skip to (3)) |
| ○Paediatrics (Please skip to (4)) |
| ○Surgery specialties (Please skip to (5)) |
| ○Obstetrics and gynaecology or subspecialty |
| ○Psychiatry |
| ○Anaesthesiology |
| ○Dermatology |
| ○Otorhinolaryngology (ear-nose-throat) |
| ○Emergency medicine |
| ○Ophthalmology (eye doctor) |
| ○Community medicine and public health/ Social and preventive medicine |
| ○Laboratory Medicine (Please skip to (6)) |
| ○Radiology |
| ○Other (please specify) _________________ |

(3) Please choose the option that you prefer most within medicine

| ○General internal medicine (primary care) |
| --- |
| ○Internal medicine or subspecialty (non-primary care) (e.g. haematology, gastroenterology, endocrinology, cardiology, etc.) |
| ○Neurology |
| ○Radiation oncology |
| ○Physical medicine and rehabilitation |
| ○Clinical pharmacology |

(4) Please choose the option that you prefer most within paediatrics

| ○General paediatrics (primary care) |
| --- |
| ○Paediatrics or subspecialty (non-primary care) (e.g. paediatric neurology, paediatric gastroenterology, paediatric endocrinology, etc) |

(5) Please choose the option that you prefer most within surgery

| ○General surgery |
| --- |
| ○Neurological surgery |
| ○Cardio-thoracic surgery |
| ○Orthopaedic surgery |
| ○Plastic and reconstructive surgery |
| ○Urology |

(6) Please choose the option that you prefer most within laboratory medicine

| ○Pathology |
| --- |
| ○Medical genetics |

1. How much do you agree/disagree that the following factors can influence your career intention? Choose 1 if you strongly disagree and 5 if you strongly agree.

| **Factors** | **Strongly disagree**  **(1)** | **Disagree**  **(2)** | **Neutral**  **(3)** | **Agree**  **(4)** | **Strongly agree**  **(5)** |
| --- | --- | --- | --- | --- | --- |
| 1. Physical condition | ○ | ○ | ○ | ○ | ○ |
| 2. Competence | ○ | ○ | ○ | ○ | ○ |
| 3. Personal interest | ○ | ○ | ○ | ○ | ○ |
| 4. Altruism (intention to help and consideration of patient’s benefit) | ○ | ○ | ○ | ○ | ○ |
| 5. Advice from family, friends or peers | ○ | ○ | ○ | ○ | ○ |
| 6. Previous or existing health problems in the family | ○ | ○ | ○ | ○ | ○ |
| 7. Teachers or mentors at school or hospital | ○ | ○ | ○ | ○ | ○ |
| 8. Studying content and environment (e.g. school curriculum, electives, clinical rotations) | ○ | ○ | ○ | ○ | ○ |
| 9. Work content and environment (e.g. job content, work pressure, autonomy, patient type) | ○ | ○ | ○ | ○ | ○ |
| 10. Role model (someone worthy of imitation) | ○ | ○ | ○ | ○ | ○ |
| 11. Work/Life balance | ○ | ○ | ○ | ○ | ○ |
| 12. Prestige (social status) | ○ | ○ | ○ | ○ | ○ |
| 13. Employment opportunities (job opportunities available in the labour market) | ○ | ○ | ○ | ○ | ○ |
| 14. Career progression outlook (e.g. career advancement, personal growth, further professional training) | ○ | ○ | ○ | ○ | ○ |
| 15. Salary/Financial reward | ○ | ○ | ○ | ○ | ○ |
| 16. Gender representation gap | ○ | ○ | ○ | ○ | ○ |
| 17. Competition | ○ | ○ | ○ | ○ | ○ |
