# Supplementary material for: Future doctors, future scholars: factors influencing China-educated international medical students’ career intentions in primary care and academic medicine
Source: Hum Resour Health. 2026 Mar 25;24:20. doi: 10.1186/s12960-026-01062-2 (PMC13137620; doi:10.1186/s12960-026-01062-2)
Supplement: Supplementary file 3 — Additional file3 (DOCX 21 KB) [file 12960_2026_1062_MOESM3_ESM.docx]

**Appendix 3** Need and perception related factors formulation and refinement

| **Source information from qualitative interviews** | **Informed by literature reviews** | **After expert validation** | **After cognitive interviews** | **After pilot study and focus group discussion** |
| --- | --- | --- | --- | --- |
| ***27 factors*** | ***25 factors*** | ***18 factors*** | ***18 factors*** | ***17 factors*** |
| Physical condition |  |  |  | Physical condition |
| Competence |  |  |  | Competence |
| Personal interest |  |  |  | Personal interest |
| Altruism |  | Combined: Personal values (Altruism and patients’ demand) | Rewording: Altruism (intention to help and consideration of patient’s benefit) | Altruism (intention to help and consideration of patient’s benefit) |
| Patients’ demand |  |  |  |  |
| Advice from family |  | Combined: Advice from family and friends | Rewording: Advice from family, friends or peers | Advice from family, friends or peers |
| Advice from friends |  |  |  |  |
| Previous or existing health problems in the family |  |  |  | Previous or existing health problems in the family |
| Clinical mentors | Combined: Teachers or mentors at school or hospital |  |  | Teachers or mentors at school or hospital |
| Teachers and faculties |  |  |  |  |
| School curriculum | Combined: Studying and environment (e.g. school curriculum, clinical rotations) | Revised: Studying content and environment (e.g. school curriculum, electives, clinical rotations) |  | Studying content and environment (e.g. school curriculum, electives, clinical rotations) |
| Clinical rotations |  |  |  |  |
| Job content |  | Combined: Work content and environment (e.g. job content, work pressure, autonomy, patient type) |  | Work content and environment (e.g. job content, work pressure, autonomy, patient type) |
| Work pressure |  |  |  |  |
| Autonomy at work |  |  |  |  |
| Patient type |  |  |  |  |
| Media influence |  | Combined: Role model (media or people around) | Rewording: Role model (someone worthy of imitation) | Role model (someone worthy of imitation) |
| Role model |  |  |  |  |
| Work/Life balance |  |  |  | Work/Life balance |
| Prestige |  |  | Rewording: Prestige (social status) | Prestige (social status) |
| Employment opportunities |  |  | Rewording: Employment opportunities (job opportunities available in the labour market) | Employment opportunities (job opportunities available in the labour market) |
| Career prospects |  | Combined: Career progression outlook (e.g. career advancement, personal growth, further professional training) |  | Career progression outlook (e.g. career advancement, personal growth, further professional training) |
| Further training |  |  |  |  |
| Salary/Financial reward |  |  |  | Salary/Financial reward |
| Gender imbalance | Revised: Gender representation gap |  |  | Gender representation gap |
| Competition |  |  |  | Competition |
| Pandemic |  |  | Suggesting deletion | Deleted |
