# Supplementary material for: Future doctors, future scholars: factors influencing China-educated international medical students’ career intentions in primary care and academic medicine
Source: Hum Resour Health. 2026 Mar 25;24:20. doi: 10.1186/s12960-026-01062-2 (PMC13137620; doi:10.1186/s12960-026-01062-2)
Supplement: Supplementary file 4 — Additional file4 (DOCX 25 KB) [file 12960_2026_1062_MOESM4_ESM.docx]

**Appendix 4-1** Comparison of factors considered by IMSs for PC preference

| **Perceived factors** | **Mean±SD** | **Choosing a primary care specialty (Mean±SD) (n=150)** | **Choosing a non-primary care specialty (Mean±SD) (n=811)** | **p-value** |
| --- | --- | --- | --- | --- |
| 1. Physical condition | 4.05±1.089 | 3.97±1.126 | 4.07±1.083 | 0.296 |
| 2. Competence | 4.12±0.976 | 4.01±1.040 | 4.14±0.963 | 0.130 |
| 3. Personal interest | 4.41±0.939 | 4.19±1.015 | 4.45±0.919 | **0.002^**^** |
| 4. Altruism (intention to help and consideration of patient’s benefit) | 4.28±0.977 | 4.15±1.090 | 4.31±0.953 | 0.067 |
| 5. Advice from family, friends or peers | 3.49±1.087 | 3.37±1.078 | 3.52±1.088 | 0.141 |
| 6. Previous or existing health problems in the family | 3.32±1.203 | 3.38±1.174 | 3.31±1.220 | 0.536 |
| 7. Teachers or mentors at school or hospital | 3.81±1.078 | 3.76±1.097 | 3.82±1.074 | 0.548 |
| 8. Studying content and environment (e.g. school curriculum, electives, clinical rotations) | 3.96±0.998 | 3.80±1.074 | 3.99±.0.981 | **0.046^*^** |
| 9. Work content and environment (e.g. job content, work pressure, autonomy, patient type) | 3.93±1.013 | 3.87±1.041 | 3.94±1.008 | 0.434 |
| 10. Role model (someone worthy of imitation) | 3.79±1.069 | 3.70±1.022 | 3.81±1.077 | 0.257 |
| 11. Work/Life balance | 4.01±0.955 | 3.95±0.951 | 4.02±0.956 | 0.409 |
| 12. Prestige (social status) | 3.71±1.119 | 3.59±1.124 | 3.73±1.117 | 0.177 |
| 13. Employment opportunities (job opportunities available in the labour market) | 3.85±1.040 | 3.76±1.034 | 3.87±1.040 | 0.227 |
| 14. Career progression outlook (e.g. career advancement, personal growth, further professional training) | 4.07±0.939 | 3.94±0.978 | 4.09±0.930 | 0.070 |
| 15. Salary/Financial reward | 3.94±1.048 | 3.78±1.042 | 3.97±1.047 | **0.037*** |
| 16. Gender representation gap | 3.25±1.243 | 3.25±1.165 | 3.25±1.257 | 0.977 |
| 17. Competition | 3.81±1.104 | 3.69±1.074 | 3.83±1.109 | 0.176 |

**Appendix 4-2** Comparison of factors considered by IMSs for AM preference

| **Perceived factors** | **Mean±SD** | **Ranking teaching /research/ leadership as top 2 options (n=349)** | **Not ranking teaching/ research/ leadership as top 2 options (n=612)** | **p-value** |
| --- | --- | --- | --- | --- |
| 1. Physical condition | 4.05±1.089 | 4.07±1.069 | 4.04±1.102 | 0.766 |
| 2. Competence | 4.12±0.976 | 4.15±0.953 | 4.09±0.988 | 0.337 |
| 3. Personal interest | 4.41±0.939 | 4.50±0.899 | 4.36±0.958 | **0.023^*^** |
| 4. Altruism (intention to help and consideration of patient’s benefit) | 4.28±0.977 | 4.30±0.893 | 4.27±1.022 | 0.621 |
| 5. Advice from family, friends or peers | 3.49±1.087 | 3.43±1.093 | 3.53±1.083 | 0.172 |
| 6. Previous or existing health problems in the family | 3.32±1.203 | 3.34±1.233 | 3.31±1.202 | 0.697 |
| 7. Teachers or mentors at school or hospital | 3.81±1.078 | 3.85±1.081 | 3.79±1.076 | 0.390 |
| 8. Studying content and environment (e.g. school curriculum, electives, clinical rotations) | 3.96±0.998 | 4.00±0.972 | 3.93±.1.012 | 0.308 |
| 9. Work content and environment (e.g. job content, work pressure, autonomy, patient type) | 3.93±1.013 | 3.94±1.011 | 3.92±1.016 | 0.752 |
| 10. Role model (someone worthy of imitation) | 3.79±1.069 | 3.81±1.074 | 3.78±1.067 | 0.661 |
| 11. Work/Life balance | 4.01±0.955 | 3.97±0.990 | 4.03±0.935 | 0.348 |
| 12. Prestige (social status) | 3.71±1.119 | 3.73±1.083 | 3.69±1.139 | 0.573 |
| 13. Employment opportunities (job opportunities available in the labour market) | 3.85±1.040 | 3.91±1.000 | 3.82±1.061 | 0.250 |
| 14. Career progression outlook (e.g. career advancement, personal growth, further professional training) | 4.07±0.939 | 4.13±0.892 | 4.03±0.963 | 0.095 |
| 15. Salary/Financial reward | 3.94±1.048 | 4.02±1.028 | 3.90±1.058 | 0.077 |
| 16. Gender representation gap | 3.25±1.243 | 3.21±1.200 | 3.28±1.267 | 0.395 |
| 17. Competition | 3.81±1.104 | 3.80±1.091 | 3.81±1.112 | 0.851 |
