# Supplementary material for: Future doctors, future scholars: factors influencing China-educated international medical students’ career intentions in primary care and academic medicine
Source: Hum Resour Health. 2026 Mar 25;24:20. doi: 10.1186/s12960-026-01062-2 (PMC13137620; doi:10.1186/s12960-026-01062-2)
Supplement: Supplementary file 5 — Additional file5 (DOCX 26 KB) [file 12960_2026_1062_MOESM5_ESM.docx]

**Appendix 5-1** SPSS results for regression Model 3 of PC specialty preference as outcome

| **Variables in the Equation** | | | | | | | | | | |
| --- | --- | --- | --- | --- | --- | --- | --- | --- | --- | --- |
|  | | B | S.E. | Wald | df | Sig. | Exp(B) | 95% C.I. for EXP(B) | |  |
|  |  |  |  |  |  |  |  | Lower | Upper |  |
| Step 1^a^ | Gender Female (REF Male) | -.213 | .193 | 1.218 | 1 | .270 | .808 | .554 | 1.180 |  |
|  | Age | .076 | .033 | 5.153 | 1 | .023 | 1.079 | 1.010 | 1.152 |  |
|  | Nationality Eastern Mediterranean Region (REF South-East Asia Region) | -.174 | .281 | .386 | 1 | .534 | .840 | .484 | 1.456 |  |
|  | Nationality African Region (REF South-East Asia Region) | .306 | .316 | .935 | 1 | .334 | 1.357 | .731 | 2.521 |  |
|  | Nationality Other (REF South-East Asia Region) | -.632 | .780 | .657 | 1 | .418 | .531 | .115 | 2.451 |  |
|  | Year of study | -.076 | .064 | 1.442 | 1 | .230 | .927 | .818 | 1.049 |  |
|  | Place of origin Regional or rural (REF Urban) | .489 | .190 | 6.659 | 1 | .010 | 1.631 | 1.125 | 2.365 |  |
|  | Doctor in family Yes (REF No) | .038 | .185 | .042 | 1 | .837 | 1.039 | .723 | 1.492 |  |
|  | Institution type by designated orientation Medicine and Pharmacy (REF Comprehensive) | .372 | .319 | 1.363 | 1 | .243 | 1.451 | .777 | 2.711 |  |
|  | Institution ranking | -.014 | .027 | .273 | 1 | .601 | .986 | .936 | 1.039 |  |
|  | Province GDP >1000 (REF <1000) | -.059 | .208 | .082 | 1 | .775 | .942 | .627 | 1.415 |  |
|  | Component 1 (Personal needs to satisfy) | -.187 | .087 | 4.662 | 1 | .031 | .829 | .700 | .983 |  |
|  | Component 2 (Perceptions of work characteristics) | -.083 | .082 | 1.009 | 1 | .315 | .921 | .783 | 1.082 |  |
|  | Component 3 (Social needs to satisfy) | .025 | .081 | .094 | 1 | .759 | 1.025 | .875 | 1.202 |  |
|  | Preferring academic medicine | -.513 | .207 | 6.137 | 1 | .013 | .599 | .399 | .898 |  |
|  | Constant | -2.497 | .995 | 6.297 | 1 | .012 | .082 |  |  |  |
|  | | | | | | | | | | |

**Appendix 5-2** SPSS results for regression Model 3 of AM career preference as outcome

| **Variables in the Equation** | | | | | | | | | |
| --- | --- | --- | --- | --- | --- | --- | --- | --- | --- |
|  | | B | S.E. | Wald | df | Sig. | Exp(B) | 95% C.I. for EXP(B) | |
|  |  |  |  |  |  |  |  | Lower | Upper |
| Step 1^a^ | Gender Female (REF Male) | -.285 | .147 | 3.772 | 1 | .052 | .752 | .564 | 1.003 |
|  | Age | .129 | .031 | 17.291 | 1 | <.001 | 1.138 | 1.071 | 1.209 |
|  | Nationality Eastern Mediterranean Region (REF South-East Asia Region) | -.007 | .220 | .001 | 1 | .974 | .993 | .645 | 1.528 |
|  | Nationality African Region (REF South-East Asia Region) | .542 | .244 | 4.922 | 1 | .027 | 1.720 | 1.065 | 2.777 |
|  | Nationality Other (REF South-East Asia Region) | .331 | .516 | .411 | 1 | .521 | 1.392 | .507 | 3.825 |
|  | Year of study | -.132 | .053 | 6.217 | 1 | .013 | .877 | .790 | .972 |
|  | Place of origin Regional or rural (REF Urban) | -.079 | .144 | .305 | 1 | .581 | .924 | .697 | 1.224 |
|  | Doctor in family Yes (REF No) | -.090 | .141 | .406 | 1 | .524 | .914 | .693 | 1.205 |
|  | Institution type by designated orientation Medicine and Pharmacy (REF Comprehensive) | -.218 | .207 | 1.103 | 1 | .294 | .804 | .536 | 1.207 |
|  | Institution ranking | -.056 | .021 | 7.248 | 1 | .007 | .946 | .908 | .985 |
|  | Province GDP >1000 (REF <1000) | .019 | .161 | .014 | 1 | .907 | 1.019 | .744 | 1.396 |
|  | Component 1 (Personal needs to satisfy) | .066 | .073 | .814 | 1 | .367 | 1.068 | .925 | 1.234 |
|  | Component 2 (Perceptions of work characteristics) | .026 | .065 | .157 | 1 | .692 | 1.026 | .904 | 1.165 |
|  | Component 3 (Social needs to satisfy) | -.049 | .061 | .637 | 1 | .425 | .952 | .844 | 1.074 |
|  | Choosing a primary care specialty | -.500 | .206 | 5.892 | 1 | .015 | .607 | .405 | .908 |
|  | Constant | -2.424 | .831 | 8.506 | 1 | .004 | .089 |  |  |
